# Software Defined Radio for GNSS Radio Frequency Interference Localization

**DOI:** 10.3390/s24010072

**Published:** 2023-12-22

**Authors:** Fred Taylor, Evan Gattis, Lucca Trapani, Dennis Akos, Sherman Lo, Todd Walter, Yu-Hsuan Chen

**Affiliations:** 1 Ann and H.J. Smead Aerospace Engineering Sciences, University of Colorado Boulder, Boulder, CO 80303, USA; frta6411@colorado.edu (F.T.); evan.gattis@colorado.edu (E.G.); lucca.trapani@colorado.edu (L.T.); 2 Department of Aeronautics and Astronautics, Stanford University, Stanford, CA 94305, USA; daedalus@stanford.edu (S.L.); twalter@stanford.edu (T.W.); shinge@stanford.edu (Y.-H.C.)

**Keywords:** global positioning system (GPS), software defined radio (SDR), radio direction finding

## Abstract

The use of radio direction finding techniques in order to identify and reject harmful interference has been a topic of discussion both past and present for signals in the GNSS bands. Advances in commercial off-the-shelf radio hardware have led to the development of new low-cost, compact, phase coherent receiver platforms such as the KrakenSDR from KrakenRF whose testing and characterization will be the primary focus of this paper. Although not specifically designed for GNSSs, the capabilities of this platform are well aligned with the needs of GNSSs. Testing results from both benchtop and in the field will be displayed which verify the KrakenSDR’s phase coherence and angle of arrival estimates to array dependent resolution bounds. Additionally, other outputs from the KrakenSDR such as received signal strength indicators and the angle of arrival confidence values show strong connections to angle of arrival estimate quality. Within this work the testing that will be primarily presented is at 900 MHz, with results presented from a government-sponsored event where the Kraken was tested at 1575.42 MHz. Finally, a discussion of calibration of active antenna arrays for angle of arrival is included as the introduction of active antenna elements used in GNSS signal collection can influence angle of arrival estimation.

## 1. Introduction

Global Navigation Satellite Systems (GNSSs) have developed into key pieces of global infrastructure, being applied for positioning, navigation, and timing (PNT) in various fields and technologies. They are critical in directing air traffic control, maritime trade, maintaining timing on electrical grids, and precision agriculture to highlight a few [1]. As such, protection of these systems has become paramount to safeguarding both civil and military industries. One of the largest threats to these systems is radio interference, which oftentimes takes the form of either jamming or spoofing. Jamming involves introducing noise power into the GNSS frequency band of interest in order to drown out the already weak GNSS signals. Spoofing is much more nefarious as it seeks to supplement one or more GNSS signals with a fake version of the signal in order to feed a receiver incorrect information and thus influence the resulting position solution. In the civil sector, occurrences of disruptive radio interference have recently been seen at Denver International Airport where a 33-h event necessitated a “Notice to Airmen” and at Dallas-Fort Worth where a similar length event caused the temporary shutdown of a runway [2,3]. The importance of quick, reliable ways to localize GNSS interference is only becoming more important as the potential threat to the integrity and accuracy of GNSSs grows.

Concerning PNT resilience, various research efforts have been undertaken to either assure the integrity and accuracy of GNSS signals or otherwise circumvent malicious interference. Current topics of research in this field include the development of algorithms or hardware capable of identifying and removing interference in GNSS receivers, machine learning, exploration of potential alternative navigation techniques for use in difficult navigation scenarios, and radio direction finding techniques. Briefly discussing these, we start with software and hardware developments to harden GNSS receivers. Various research efforts have been undertaken to explore this field. Techniques involve adaptive notch filtering, empirical mode decomposition, wavelet filters, zero-memory non-linearity, pulse blanking, and fast Fourier transform filters [4,5,6]. These techniques excel at narrowband interference detection and exclusion and often help to isolate GNSS signal from interference to assist in ensuring position and timing accuracy by removal of suspected interference in the data [4]. Where these techniques lack is that they are often not robust, with each being suited for specific types of GNSS interference waveforms. Additionally, they can become very hardware or computationally demanding making them unsuitable for low-cost, compact receivers [4,5]. Machine learning algorithms are similar to the previously mentioned techniques in function. By applying machine learning methods to GNSS problems, generalized solutions can be obtained for not just interference, but also topics such as acquisition, precising positioning, and atmospheric effects [7]. Like the previously discussed software and hardware techniques, machine learning has shown improvement over standard models and results. Despite this, they suffer from several of the same drawbacks as other software and hardware techniques. Different machine learning models are suited for different applications, so the proper model choice is paramount to seeing improvement [7]. Additionally, sufficient computational resources must be available to run the model along with requiring training data, which may not be readily available for all scenarios such as jamming or spoofing. Next, a common topic for potential interference mitigation is alternative navigation. This topic generally focuses on increasing the overall number of signals available or setting up separate systems and signals capable of obtaining PNT solutions [8,9,10]. These systems offer the capability to navigate even in GNSS-denied environments as they often are designed at other operating frequencies and may, with correct receiver setup, improve GNSS position estimates during healthy operation. Currently, there are two major drawbacks to these systems. The first is that their accuracy is generally very coarse, as it is seen when working with DME positioning or the LEO constellation Iridium, which both have position errors exceeding 150 m [9,10]. Second is that for widespread application PNT receiver architecture would need to be overhauled to allow for acquisition of these signals. This would be expensive both monetarily and temporally, limiting alternative navigation system potential. Finally, research into radio direction finding techniques, which will be the focus of this paper, has been a longstanding area of interest [11,12,13,14,15]. Commonly, these techniques involve the computation of a signal source angle of arrival (AoA), which is then used to drive antenna array beam patterns through beamforming and/or nulling [11,14]. These systems have a wide case of applications, not being inherently limited by waveform type, and through AoA estimation and beamforming methods can reject unwanted signals while improving the signal strength of desired signals [12,13,15]. One of the prohibitive aspects of radio direction of arrival application though has been its reliance on expensive, complex hardware, which made widespread adoption infeasible.

Outside of radio direction finding, one major issue that the other techniques discussed face is that they are not capable of directly dealing with interference. Software and hardware interference detection algorithms, machine learning, and alternative navigation each seek to mitigate the interference by circumventing its impact through either removing it or working around it. As such, these techniques currently do not restore healthy GNSS operations to an area. Billions of dollars have been invested in GNSS systems, so restoring healthy operations to enable PNT maximizes the ability to leverage them [16]. Furthermore, for widespread adoption of any of these three techniques, the current receiver architecture would need to be considerably modified, taking large investments of time and money. With this in mind, the ability to localize interference by estimating an emitter location would be ideal as it allows for healthy GNSS operations to be recovered. Radio direction finding provides this opportunity as the intersection of multiple AoA bearings could be used to locate a signal source. With the continued improvement of software defined radio (SDR) hardware along with research in this field, compact, cost-effective radio platforms have been developed that overcome the previous hurdle of expensive and complex hardware limiting widespread use [17]. One such product is the KrakenSDR developed by KrakenRF (Chicago, IL, USA) [18]. This commercial off-the-shelf (COTS) SDR receiver platform became available for general purchase in 2021 after a successful crowdfunding campaign. Capable of spanning a frequency band of 24 MHz to 1766 MHz and supporting up to five antenna elements, the KrakenSDR is perfectly suited for use in radio direction finding [18]. Focusing this on the GNSS bands, which range from about 1000 MHz to 1700 MHz, has the potential to apply this cost-effective hardware in a novel, impactful way. The focus of this paper will be to present, characterize, and test the KrakenSDR hardware in the open industrial, scientific, and medical (ISM) band before assessing initial testing carried out in the L-band, which will require additional calibration efforts, where GNSS signals reside. To collect AoA estimates from the KrakenSDR, the built-in algorithms and data collection functions can be leveraged to record AoA as well as associated signal and estimate metrics. Comparison to expected truth values based on array layout and a known signal source location in combination with estimate metrics allow for performance to be assessed. Additionally, comparing statistics of the results to array and radio direction finding algorithm dependent resolution bounds provide further insight into KrakenSDR’s performance.

Within this paper, we will explore the features and functionality of the KrakenSDR to showcase its potential as a compact, easy-to-use tool for emitter localization. A brief discussion of the hardware itself, both the KrakenSDR and utilized antenna arrays, as well as the calibration and AoA estimation software will first be performed before moving into testing. Testing is divided into four subsections, with each outlining the process and results of a set of tests carried out on one aspect of the KrakenSDR’s functionality. The experiments presented will include an exploration of the KrakenSDR’s internal calibration, a subset of AoA tests performed at 900.1 MHz, a brief look at its potential for making AoA estimates on multiple signal sources at 900.1 MHz, and then finally a set of initial tests in the L-band. These experiments only highlight a fraction of the potential utility of the KrakenSDR. Following this, a discussion of future work will be presented before finally ending with a conclusion to summarize the paper’s content.

## 2. Materials and Methods

**Figure 1:**
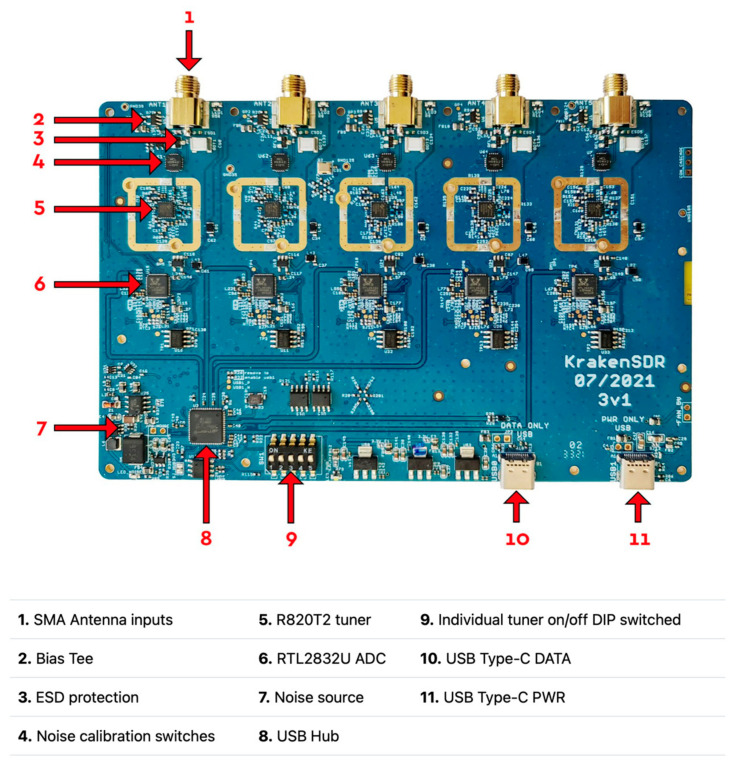
Labeled printed circuit board of KrakenSDR provided by KrakenRF [19].

As was mentioned in the introduction, the KrakenSDR is the primary focus of exploration within this work. The KrakenSDR is comprised of not just the physical hardware platform including the five RTL-SDR receivers, common clock, and noise generator but also the open-source software provided by KrakenRF [18,19]. This open-source software has two primary components, being the data acquisition (DAQ) and the digital signal processing (DSP), which collect data and process the data using direction-finding algorithms, respectively [18]. The physical platform is 500 USD, which represents a relatively inexpensive cost for its value in conjunction with its free open-source software. Spanning the range of 24–1766 MHz while having a maximum bandwidth of 2.56 MHz additionally makes the hardware ideal for capturing many but not all of the GNSS signals. The full technical specifications provided by KrakenRF are displayed in Table 1 and an image of the KrakenSDR’s printed circuit board can be seen in Figure 1.

In addition to the KrakenSDR itself, two different antenna arrays had to be designed in order to provide the KrakenSDR with data capable of computing signal AoA in its algorithms. The KrakenSDR is capable of supporting uniform linear arrays (ULAs), uniform circular arrays (UCAs), and custom array configurations. This paper will present the two UCA setups utilized to produce the results seen later. A UCA was chosen for two primary reasons. First, the KrakenSDR natively supports and has been designed with UCA arrays in mind, making it easy to compute proper array dimensions through the use of a provided “Antenna Array Calculator” tool [20]. Secondly, as opposed to a ULA, there is no ambiguity in the direction of the incoming signal due to array layout meaning you achieve full 360-degree coverage for a UCA as opposed to 180-degree coverage for a ULA. For both a ULA and UCA it is desirable to have equal interelement spacing where the interelement spacing, *I_e_*, is defined by Equation (1).

*I_e_* = *s*λ (1)

Within Equation (1), λ is the wavelength of the desired signal frequency and *s* is a nonnegative multiplier less than or equal to 0.5. When defining *s*, a couple of elements must be kept in mind based on the desired operation. First, *s* must be less than or equal to 0.5 because if *s* is greater than 0.5 then the interelement spacing is greater than half a wavelength and your array will have grating lobes, which lead to ambiguities in signal AoA computations. Second, values of *s* close to 0.5 provide the best resolution for AoA estimates, at about 8-degree arc resolution, so although reducing *s* will help to limit the size of the antenna array being designed it is a trade-off for potential accuracy of the solution. For example, reducing *s* to 0.4, 0.3, or 0.2 will reduce resolution to about 10 degrees, 14 degrees, or 25 degrees, respectively, for a given frequency. A quick aside: the arc resolution is computed from the Rayleigh criterion as defined in Equation (2) from wavelength and array diameter. This value is then divided by a common factor of 10 for super-resolution algorithms, like the one leveraged in this paper, to obtain the values of arc resolution referenced throughout this work.

(2) $$\theta \;[\mathrm{radians}]=\sin^{-1}(1.22\frac{\lambda }{D})$$

**Figure 2:**
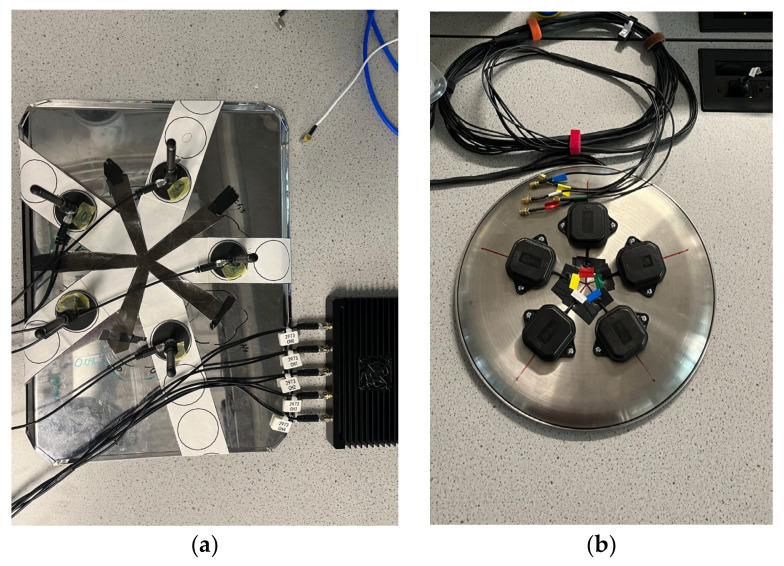
Antenna arrays used for testing within this paper: (**a**) 900 MHz array (**b**) L-band array.

The two arrays used in this work are a 900 MHz passive antenna array and L-band active antenna array, both with five elements and with parameters described in Table 2 and physically shown in Figure 2. Of note is that the L-band array is designed around a frequency of 1600 MHz, which provides the array with a multiplier, *s*, greater than 0.3 for all frequencies greater than 1000 MHz. This corresponds to AoA arc resolutions of between about 8 degrees at 1600 MHz to about 13 degrees at 1000 MHz. The antennas used for the 900 MHz array were the magnetic whip antennas offered by KrakenRF, while the antennas used for the L-band array were u-blox ANN-MB-00 multiband GNSS antenna, which required the bias tee on the KrakenSDR to be enabled.

An important aspect of the KrakenSDR that warrants discussion is its calibration. Each of the five RTL-SDRs utilized by the KrakenSDR is its own channel but in order to make proper AoA estimates they must all be synced and kept phase coherent. To achieve this the KrakenSDR uses a common clock for all five SDRs, but it also leverages a noise source and calibration software to achieve phase coherence by quantifying and accounting for the phase offset between the channels upon startup. The calibration is achieved in the DAQ side of the KrakenRF software. Two finite-state machines (FSMs) handle the phase and gain calibrations separately. Since the KrakenSDR uses an eight-bit ADC, the signal could be quantized if the gain is not set properly. In order to prevent this, the gain FSM operates to maximize each channel’s gain independently without causing saturation of the signal. On each channel, the gain is increased up until the point of signal saturation, and then decreased slightly once that point is reached.

**Figure 3:**
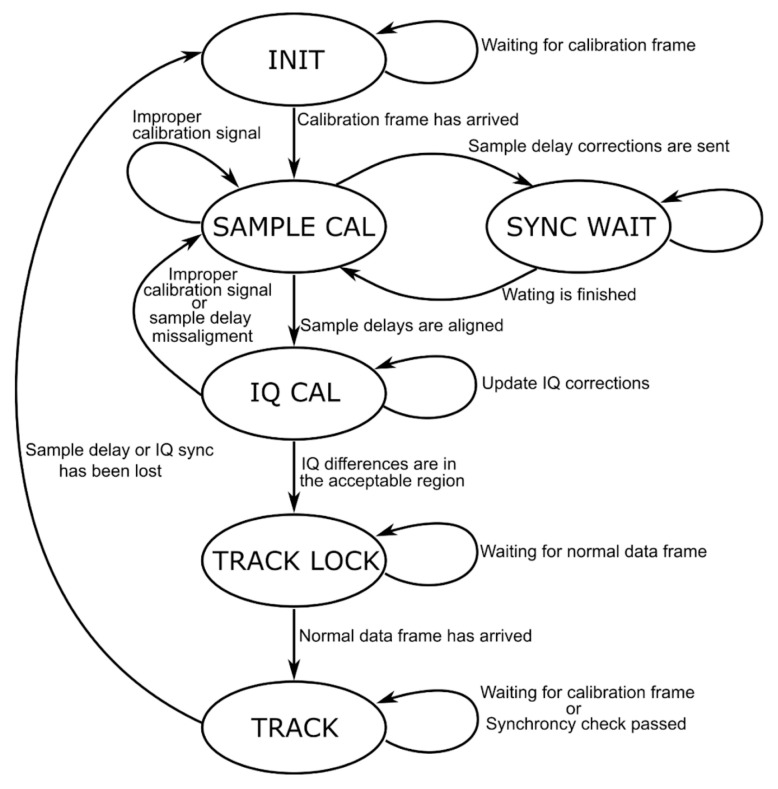
Sample alignment process included in KrakenSDR documentation [21].

The second “Delay Synchronizer” FSM handles the phase-coherence. First, all *M* channels must be sample aligned. The FSM estimates each channel’s fractional sample delay from the base channel using the phase-frequency difference curve of the channels. The fractional time delays are directly related to the slope of this line. The DAQ uses these sample delays to issue corrections to the front-end receivers. Once the channels are sample-aligned, the FSM moves on and uses the amplitude and phase differences between channels to issue IQ corrections. The incoming IQ samples are simply multiplied by the complex corrections. Once the phase delays and amplitude differences are within configurable bounds, the FSM enters track mode. In track mode, the FSM only checks for synchronization, and does not issue any new corrections unless the estimated synchrony is outside the bounds. Once the FSM reaches this state, the IQ samples are marked as calibrated and are processed in the DSP side of the software. This process is summarized in Figure 3 from the KrakenSDR documentation. It is important to recognize that this internal calibration cannot account for interchannel biases within the typical active antenna design, with its filters and amplifiers, most popular for GNSSs.

Finally, a brief presentation of the AoA estimation technique used will be provided. The KrakenSDR is capable of multiple different AoA estimation methods, including but not limited to Bartlett, Capon, and MEMS. For this paper, the super-resolution Multiple Signal Classification (MUSIC) algorithm was leveraged. The MUSIC algorithm is a well-documented and explored subspace technique having spawned various offshoot algorithms which provides very high angular resolution while also working well for low SNR signals [22,23]. It assumes the noise subspace and signal subspace are orthogonal and can be computed through eigen decomposition of the sensor sample covariance matrix derived from the received signal. Additionally, it is also capable of identifying multiple sources given a priori knowledge of the number of sources, which could be desirable in future applications. For this paper, the MUSIC algorithm computes estimates at about a 1 Hz rate over data collected at 2.56 Msps. These settings are adjustable by the user. Within the MUSIC algorithm, the confidence value is computed by taking the ratio of the peak power of the MUSIC spectrum by the mean, or essentially “noise” level of the MUSIC spectrum.

## 3. Results

### 3.1. Phase Coherence Testing

**Figure 4:**
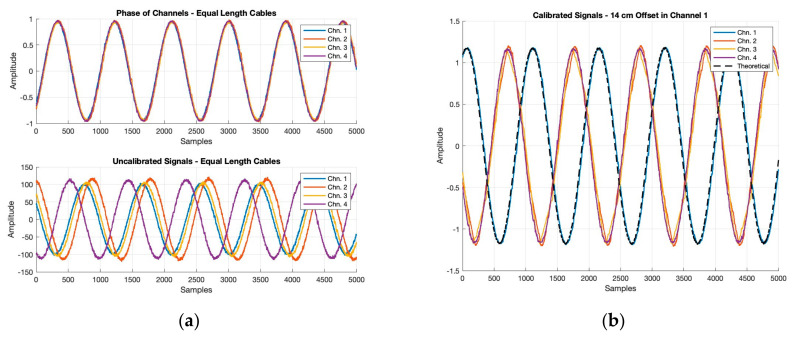
Real-world and theoretical results of phase coherence testing: (**a**) top: calibrated sine waves recorded by KrakenSDR channels using equal length cables; bottom: uncalibrated sine waves recorded by KrakenSDR channels using equal length cables; (**b**) calibrated sine waves recorded by KrakenSDR channels with 14 cm offset at channel 1 as well as the theoretical offset sine wave expected using common signal speed approximation through coaxial cable.

Before field testing, an initial exploration and confirmation of the calibration conducted within the KrakenSDR was desired. The calibration is a key aspect of the KrakenSDR as without it the AoA estimates would be biased. In order to test this, a benchtop experiment was performed. Using a signal generator, a simple sine wave at 900.1 MHz was generated and passed over cables with an equal length of 31 cm to four channels of the KrakenSDR using a signal splitter. Plotting the resulting sine wave seen at each of the channels after calibration had been performed produced the results seen in the top subplot of Figure 4a. This figure shows that all channels post-calibration recorded nearly equivalent sine waves, having corrected any phase offsets and normalizing the amplitude. To highlight the impact of this process, the bottom subplot of Figure 4b shows the uncalibrated signals arriving at the KrakenSDR. Table 3 has been included to show the phase offsets between the channels for both the calibrated and uncalibrated data. These values emphasize the significance and importance of the calibration as the phase differences are reduced by an order of magnitude between all channels. This will ensure strong AoA estimates by reducing the threat that internal phase offsets when combined with signal arrival offsets skew measurements in a way that results in incorrect AoA estimation. Before calibration, the phase offsets of the channels range from 15.68 degrees to 137.97 degrees, which corresponds to about 0.14λ to 0.38λ wavelength offsets. This is improved to between 1.67 and 6.94 degrees post-calibration corresponding to wavelength offsets between 0.005λ and 0.02λ. The standard deviations for both the calibrated and uncalibrated are close in value, being generally around 1 to 2 degrees but an improvement to these values is seen post-calibration as the values decrease for all channels by about 0.5 degrees. This would indicate less variation in phase difference between channels post-calibration.

Comparing these results to previous work carried out by other research groups, the KrakenSDR internal calibration can further be validated. One very recent study by scholars from the University of Edinburgh looked at using a self-calibrating circuit to achieve phase synchronization [24]. This technique was capable of bringing the phase mismatch between two channels to within approximately 1.5 degrees. Another group working for the Mitsubishi Electric Corporation based out of Japan developed a method similar to the rotating element electric field method (REV), which had a maximum phase difference of 3.06 degrees [25]. Finally, a group composed of individuals out of universities from Turkey, Iran, and the United Kingdom proposed a novel two-layer Butler matrix, which had phase error less than 5 degrees [26]. Comparing these values to the 4.16 degrees average achieved by the KrakenSDR, we can immediately see that phase errors achieved by the KrakenSDR are on the same order of magnitude. The average phase error is less than the two-layer Butler matrix, which is a variation of the Butler matrix commonly used in beamsteering, by about a degree while it is larger than the phase synchronization circuit and modified REV by about 2.5 degrees and 1 degree respectively. The advantage of the KrakenSDR’s method is its simplicity and how it has already been implemented in a compact fashion to calibrate up to five elements simultaneously. The modified REV method requires a vector network analyzer to directly, and simultaneously measure the electric field at multiple elements. It was found that the measurement error degrades with the number of elements being simultaneously shifted, so it trades off speed for accuracy with only up to three simultaneous elements shifted in the paper [25]. For the self-calibrating circuit this research is still very much in the prototype stage and is thus not compact or easily portable like the KrakenSDR. Additionally, phase difference results were only presented between two elements.

The other test performed to explore the KrakenSDR phase calibration involved introducing a 14 cm cable offset to channel 1, creating a total path length to channel 1 of 45 cm. The results for this case are shown in Figure 4b. It is now evident in Figure 4b that one of the sine waves trails the others. This is expected, because the increased path length means that the first channel’s signal will arrive later than the others, and thus be at a different phase. A theoretical model and plot were also created to verify that our result was to be expected. This was carried out by simulating a sine wave at 900.1 MHz. Using an oscilloscope, it was possible to approximate the speed of signal propagation through the extended cable we used in testing. The delay through the cable was measured at 2.26 nanoseconds around 900.1 MHz, which in combination with knowing the cable length allowed for the speed of propagation in the cable to be estimated as 0.6642 times the speed of light. The phase offset due to the additional 14 cm cable length was then estimated as 132.17 degrees. Figure 4b includes this expected theoretical signal as a black dashed line, which is seen to be overlayed on the solid blue line representing the sine wave at channel 1. The estimated phase lag of the theoretical and actual signal with respect to the other channels is presented in Table 4. From this table it can be seen that the experimental results are very close to the model that was set up. The standard deviations are on the same order of magnitude being between 1 to 2 degrees and the mean values are also very close. Of note is that the experimental results are all approximately 5.7 degrees larger than theoretical results. One thing the theoretical model does not take into account is the channel phase offsets that we see at the KrakenSDR frontend. These offsets, which we saw could be in the range of 2 to 7 degrees, encapsulate the error between the experimental and theoretical phase offsets. The results presented in this section confirmed the expected behavior of the KrakenSDR calibration, and give us confidence that any phase differences seen are primarily due to differences in signal arrival at array elements, allowing for precise and accurate AoA measurements.

### 3.2. 900 MHz DOA Testing

**Figure 5:**
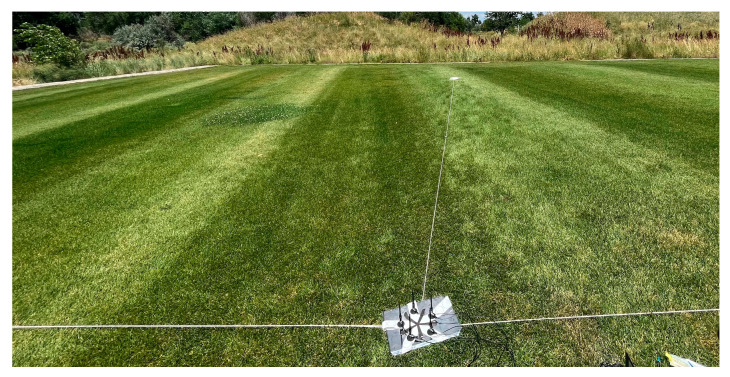
Physical setup of antenna array for single and multiple 900 MHz testing.

With confirmation of phase coherence between KrakenSDR channels, field testing was performed to explore the accuracy of its AoA estimates. This field testing was performed using the 900 MHz array described above. Although the desired implementation is for GNSSs, testing live over-the-air RF emissions within that band is cumbersome, requiring levels of authorization. The 900 MHz Industrial, Scientific, and Medical (ISM) band allows live emissions testing with minimal oversite and regulation. In order to test the MUSIC algorithm built within the software the 900 MHz array was placed at the center of a set of axes created in a field. This set of axes was created using string, pins, and measuring tools to try and ensure 90-degree differences between axes lines. With the array placed at the center and the physical axes setup, two different types of tests were performed. The first was a single emitter test where the emitter was placed at each of the axes aligned angles of 0, 90, 180, and 270 degrees with respect to the array reference antenna. The second sought to leverage the ability of MUSIC to estimate multiple sources and had two emitters placed along two separate axes 90 degrees apart. For both the single emitter and multiple emitter test a Spartant RF wideband radio frequency signal generator (Ettus Research, Austin, TX, USA) outputting a continuous 10 dBm wave at 900 MHz was used. For the multiple axes test, the second emitter used was an Ettus b200 USRP SDR transmitting a custom 10 dBm GPS-like modulated signal at 900 MHz. Of note is that pins were placed along each axis 11.5 feet, or just over 10 wavelengths, away from the center to minimize the difference in test setup when placing an emitter along an axis. A picture of the physical setup is provided in Figure 5.

#### 3.2.1. Single Emitter

**Figure 6:**
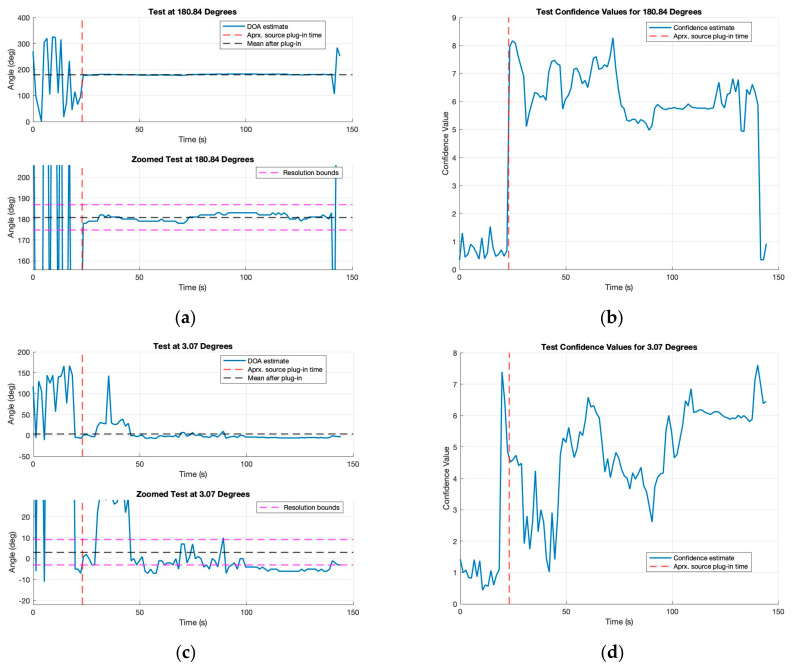
AoA or DOA calculations and their confidence values over time for two tests: (**a**) 180-degree test for AoA estimates; (**b**) 180-degree confidence values; (**c**) 0-degree test for AoA estimates [16]; (**d**) 0-degree confidence values [17].

As mentioned, the single emitter test was performed by placing the emitter at the angles or 0, 90, 180, and 270 degrees with respect to the reference antenna of the antenna array. In each of the single emitter tests, the same procedure was used. First, the KrakenSDR was turned on and then an indicator provided by the GUI was used to determine once calibration had been completed. Following this, the data collection was started without the emitter on. After 20 s of “clean” data, the emitter was then turned on and the data were collected for about a minute and half. Presented in Figure 6 are the results from the test around 180 degrees and the test around 0 degrees.

The plots presented in Figure 6 are a subset of the four tests performed. Figure 6a,c show the AoA estimates from the MUSIC algorithm as blue lines, as well as the approximate emitter turn on time about 20 s into the file as the red dashed line. Additionally, these plots show the mean value after emitter activation as a black dashed line. In the zoomed plots, the pink dashed lines represent the ±6-degree resolution bounds determined by the array design. In Figure 6b,d the confidence values computed during the MUSIC algorithm are shown, with the line for approximate emitter activation also included.

First, looking at the 180 degree plots, the AoA estimates look very close to the expected values. In the 20 s period before emitter activation, the KrakenSDR does not provide any consistent AoA estimate, which is expected as there is no signal source actively transmitting. Following emitter activation, a distinct change in behavior is seen. The value of AoA now has a mean of 180.8 degrees and the variations as well as the expected truth value of 180 degrees fall in the desired and expected ±6-degree resolution bounds. To analyze the confidence values in Figure 6b, we can compare them with the AoA estimates. As expected, the confidence is low during the erratic, no-emitter time frame of the plot. However, when the emitter is turned on 20 s into the test, the confidence value rises up from about 1 to 6. This indicates a well-defined peak in the MUSIC spectrum. Looking at the second set of plots Figure 6c,d, the results are worse than for the original, especially for the first minute after emitter plug-in. Exactly why these results are poorer in the first minute after emitter activation is uncertain, but it is theorized that a calibration issue may have occurred as this was the first test performed after powering the KrakenSDR on. These poor AoA estimates are reflected in the confidence values. At around 45 s the confidence dips back down to around 1. This indicates that there was not a distinguishable peak in the MUSIC spectrum at this time. This behavior was unable to be replicated again but it does provide an indication that using the confidence value in tandem with AoA estimates could allow any algorithms utilizing the AoA to adjust their trust accordingly.

The mean and standard deviations of AoA and confidence values for each of the four tested angles are provided in Table 5. These values were computed by taking the angle of arrival estimates in the range of 23 to 135 s into the files. This ensured the values were computed after the emitter was turned on but also did not include any values near the end of the file, where the emitter may have been deactivated before stopping data collection. Within this table all of the tests have mean values whose ±6 degrees arc resolution encapsulate the expected truth value over the minute and half collection period, even the 0-degree test with the erroneous results. Over all four tests, the combined average offset from each of their respective truths was estimated to be about 2.475 degrees. The largest average offset from the truth is seen in the 90-degree test and has a value of 3.79 degrees while the smallest offset from truth was seen in the 180-degree test at 0.83 degrees. These values indicate that the KrakenSDR makes consistent estimates over its angular range with the offsets themselves being most likely due to slight errors in setup as achieving perfect angular alignment is difficult between the array and transmitter. Of note is that the total average offset is less than half the arc-resolution, indicating the KrakenSDR performs better than its theoretical bounds. To further emphasize the consistency of the results, we can look at the standard deviations. All but the 0-degree test, which was identified as containing abnormal behavior, have 1 and 2-σ standard deviations within ±6 degrees, indicating that 95% of all estimates fall within the desired resolution arc. These results indicate that across time the KrakenSDR estimates stay consistent to within about 2 degrees meaning that it could be possible to leverage not only individual AoA estimates to locate signals but also averages of those estimates over time, like what is carried out here. Confidence values reflect the results discussed for the AoA values. The lowest average confidence value is seen during the 0-degree test, which is consistent with the results discussed previously in Figure 6b,d. The highest average confidence value is seen in the 90-degree test being larger than both the 6.29 and 6.10 values associated with the 180 and 270-degree tests. Of interest is that when comparing the standard deviations of the confidence and AoA values, larger standard deviations in one value correlate directly to larger standard deviations in the other. Intuitively this makes sense as if you are more uncertain in the AoA estimate and thus expect more variation; this should manifest as uncertainty in your confidence as both values are estimated from the MUSIC spectrum. The results of this section validate that the KrakenSDR can make consistent high accuracy AoA estimates.

#### 3.2.2. Multiple Emitters

The multiple emitter test results have to be presented in a slightly different format to the single emitter tests because the KrakenSDR only directly reports the best MUSIC estimate. The multiple emitter test was run in two formats. The first involved only searching for one source while two emitters were on and the second involved searching for two sources with two emitters on. For both of these experiments the Spartant RF was placed at the 0-degree point while the b200 was placed at the 270-degree point.

**Figure 7:**
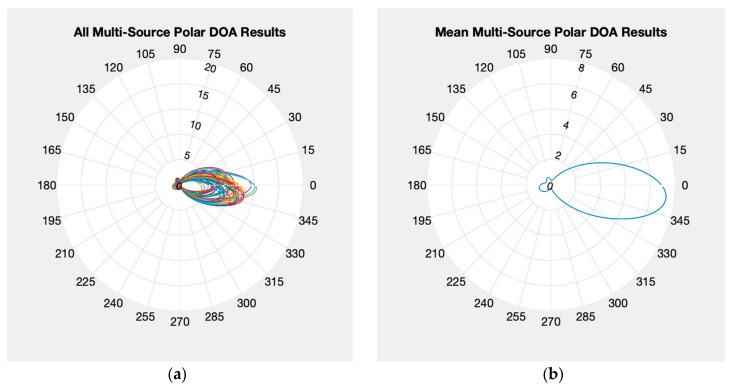
Multi-source polar plots for two emitters with single expected source: (**a**) individual solutions; (**b**) mean solutions.

Presented in Figure 7 are the results for the first style of test. In Figure 7a is the polar AoA results for each measurement epoch overlayed on top of each other and in Figure 7b is the mean of all these measurement epochs. Both results indicate that the KrakenSDR MUSIC algorithm result favored the Spartant over the b200. This makes sense as although the Spartant and b200 were setup to output the same level of power, previous testing with a spectrum analyzer using the b200 has shown it outputs are slightly under the expected production specifications. Combine this with only having control of a gain term for the b200, not the actual transmit power, and this explains why the Spartant, which does offer direct control of the output power, was dominant in this experiment. Even with the results favoring the Spartant, the result is slightly skewed toward the 270-degree direction. This indicates that if the KrakenSDR is configured to look for too few sources, any additional sources could skew some of the results from truth. Despite this behavior, the confidence values were relatively strong at around 6 on average for this testing setup.

**Figure 8:**
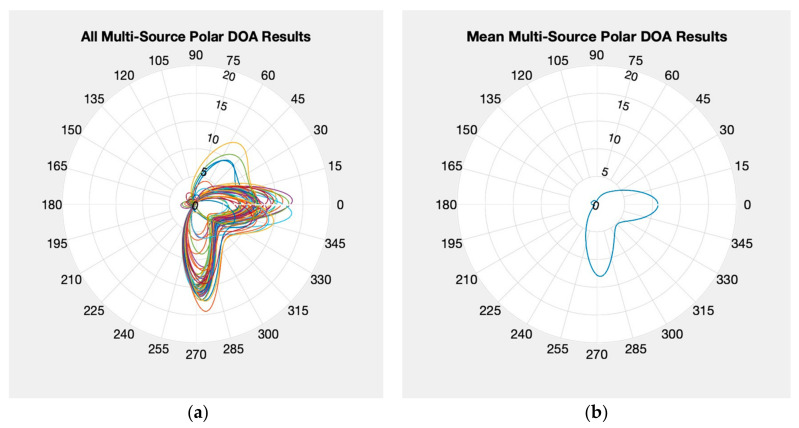
Multi-source polar plots for two emitters with two expected sources: (**a**) individual solutions; (**b**) mean solutions.

Now we move on to the multiple source test where the KrakenSDR is configured to search for the correct number of sources (two). This test is shown in Figure 8, which looks distinctly different to Figure 6. There are now two noticeable lobes in Figure 8a, which when averaged in Figure 8b indicate a source near 0 degrees and near 270 degrees, which is expected. The confidence value that the Kraken reported had also increased to an average of about 7 for this test. The individual multi-source solutions seem to indicate that for several epochs there were erroneous estimates but the resulting average over the entire minute time frame produces very promising results. Of note is that the environment tested in was in close proximity to the Aerospace Engineering building at University of Colorado, Boulder. This means that the errors seen in the plots may be due to the multipath from the building in the 90-degree direction. Additional testing is being conducted to assess how the actual vs. expected number of sources impact the resulting KrakenSDR outputs. However, these results show that the KrakenSDR can make AoA measurements of multiple signals with the proper settings.

### 3.3. 1575.42 MHz DOA Testing

**Figure 9:**
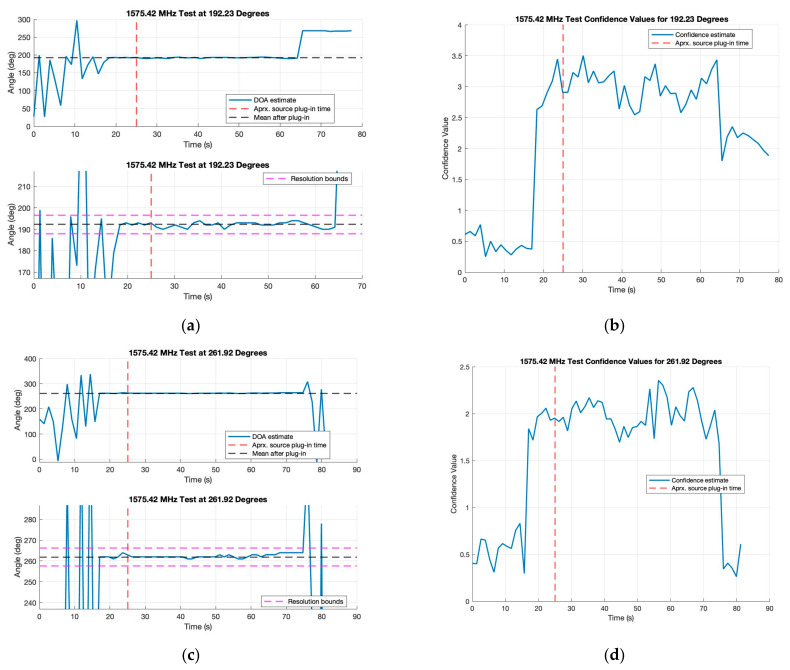
AoA or DOA calculations and their confidence values over time for two tests at L1 bands: (**a**) 180-degree test for AoA estimates; (**b**) 180-degree confidence values; (**c**) 270-degree test for AoA estimates; (**d**) 270-degree confidence values.

Continuing from the 900 MHz testing, an opportunity arose where it was possible to test the KrakenSDR using live L-band interference at a government-sponsored event. This testing took a similar format to the single emitter test, with a GNSS L1 jammer emitting at 1575.42 MHz approximately 10–15 feet from the L1-band antenna array. Emitting at 1575.42 MHz, this jammer will interfere with not only GPS L1 but also Galileo E1 and Beidou B1C. Extensive test setup verification could not be performed because the KrakenSDR was not the main focus of the testing at this event. Regardless, the results have been interesting, specifically in informing future research. The results in Figure 9 show several solutions from the same tested angles as presented in Table 5. All values are organized in Table 6.

Immediately what stands out from these plots is that the “true” values do not appear to be within the approximate 8.5-degree resolution arc for this array at 1575.42 MHz. More discussion of this will be performed later, but it is theorized that this is most likely due to the use of active antenna elements in the L-band array, which will introduce phase and gain issues not accounted for in the KrakenSDR radio calibration. These will manifest in the MUSIC algorithm as offsets to the final AoA estimates. However, even with a non-calibrated array, the angle estimates for the 180- and 270-degree tests are within 10 degrees. The 0-degree test is about 25 degrees off, which is less promising, and the 90-degree test was severely off target. These results are most likely due to the rushed nature of the test setup, as the emitter was placed very close to a vehicle for these tests. This setup may have caused a skew to the bearing angle. The confidence values were likely being influenced by very close (in-phase) reflections from the car.

## 4. Discussion

The results presented in the prior section show that there is potential for the use of the KrakenSDR in the L-band as a low-cost compact SDR platform. The results at 900 MHz indicate that a COTS phase-coherent antenna array system is adequate for estimating AoA values. These estimates not only fall within expected resolution bounds, but simple confidence metrics are a strong indicator of the quality of the estimates themselves. The compact nature of the KrakenSDR allows it to be easily relocated and tested while working within all necessary and theoretical tolerances. Continued exploration into these arising coherent receiver platforms for various applications seems warranted considering the advancements they could allow for signal source identification.

**Figure 10:**
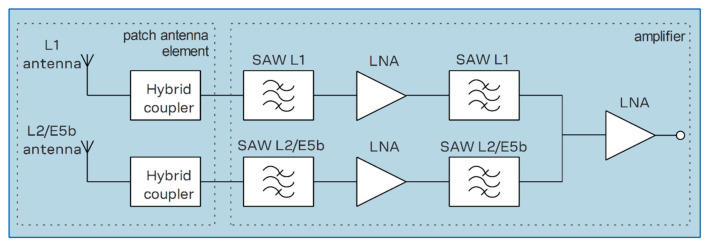
Block diagram of u-blox ANN-MB-00 multiband GNSS antenna [30].

Although direct results at 1575.42 MHz leave some room for improvement, this guides future research on the topic. Future work would primarily be focused on the calibration of an active antenna array because array calibration becomes much more important when transitioning from passive to active elements. Components within an active antenna such as amplifiers and filters introduce their own phase and gain offsets to incoming signals. The block diagram for the u-blox ANN-MB-00 multiband GNSS antenna used in this testing is presented in Figure 10 as an example of how active antenna elements are constructed. For an antenna array, this means that even if the radios are kept phase coherent, as the KrakenSDR does, the active elements will introduce erroneous phase and gain offsets that bias any AoA estimates. The largest difficulty with this is that calibrating antenna arrays has historically been difficult to do in situ because it requires signals with known arrival directions and various signals from different arrival directions to compute comprehensive calibration coefficients [27]. Recent research has focused around using GNSS signals, whose AoA measurements can be estimated in various ways, in order to compute the calibration coefficients of an antenna array [27,28,29]. These techniques generally update the antenna array model given in Equation (3) where **y**(**t**) = [y_1_(t),…, y_M_(t)]^T^ is the observed signal at an M element array, **s**(**t**) = [s_1_(t),…, s_L_(t)]^T^ contains the L present signals, **A**(**φ**,**θ**) is the M × L steering matrix built from the steering vectors related to each signal, and **n**(**t**) is the M × 1 noise [27].

**y**(**t**) = **A**(**φ**,**θ**)**s**(**t**) + **n**(**t**) (3)

There are several ways presented in current literature to update this model. In one such technique by Yang and Soloviev, two large sources of potential active array errors are calibrated. For mutual coupling, which models how antennas in close proximity influence each other, an M × M matrix, **M**, is introduced. Phase and gain mismatch caused by active elements is then modeled by a M × M diagonal matrix **D**. Introducing these matrices to Equation (3) results in Equation (4) [28].

**y**(**t**) = **MDA**(**φ**,**θ**)**s**(**t**) + **n**(**t**) (4)

From this updated form of the array, it is then possible to iteratively solve for the calibration coefficients in **M** and **D** provided a known signal azimuth and elevation of arrival by adjusting the cost function leveraged by the MUSIC algorithm [28]. A more complex model is presented by Zorn et al. This model additionally accounts for crosstalk uncertainties and explicitly divides the phase and gain mismatch parameters into stable and time-varying, before iteratively solving for the parameters using the Levenberg–Marquardt method [29]. Both of these processes exploit other methods to obtain GNSS satellite azimuth and elevation angles to deal with the problem of known signal direction of arrival. Leveraging these or other methods currently available in the literature would be a powerful technique. An antenna array could be setup in an area where GNSS integrity is important, and then compute calibration coefficients from known GNSS signals in the sky. Then, using the coefficients, it could detect AoA from any interference it detects. Combining this idea with a low-cost COTS platform like the KrakenSDR could produce a widely available system for signal interference localization.

Additional research may also look to fuse the AoA estimates of platforms like the KrakenSDR with other signal localization techniques, such as power difference of arrival or time difference of arrival. Combining the results of multiple signal source localization techniques could create a more robust system, capable of high accuracy signal source localization. Each of these techniques has their strengths and weaknesses, so combining them may allow for those weaknesses to be mitigated.

## 5. Conclusions

In conclusion, the KrakenSDR represents a powerful piece of hardware relative to the cost that one can acquire it for. The KrakenSDR is a new software-defined receiver platform which presents a novel advancement in radio direction finding through its wide range of functionality and small-scale form. One specific application it could excel at is use in the GNSS frequency band to detect and localize interference as its operable frequency range is 24 MHz to 1766 MHz. Unlike many other anti-interference techniques currently popular in both application and research, the use of AoA measurements presents the opportunity localize interference sources to restore healthy GNSS operations to a region without necessitating widescale receiver architecture changes. With that goal in mind, we desired to analyze the functionality of the KrakenSDR first in the ISM band before applying it to the L-band where GNSS signals reside.

Before testing the AoA capabilities of the KrakenSDR, an exploration of its internal phase calibration was performed. This calibration component is what enables the KrakenSDR to compute its AoA measurements accurately, so characterizing it was crucial. Benchtop testing with a known signal source was performed where the signal path length into each channel could be controlled via cable lengths. Three different types of data were collected for this analysis. Uncalibrated data, calibrated data with equal length cables connecting the KrakenSDR channels to the signal source, and calibrated data where a cable offset was introduced to one channel. Before calibration the average interchannel phase offset was over 70 degrees while post-calibration the average interchannel phase offset was 4.16 degrees. This massive improvement is seen across all of the channels and is in-line with other methods discussed. When the cable offset was introduced the results for the data collected from the KrakenSDR produced a phase offset of 137.42 degrees, which was within 6 degrees of a theoretical model developed. These tests validated that the KrakenSDR can confidently expect accurate AoA measurements due to measured phase offsets being predominantly dependent on signal path before entering the KrakenSDR.

To explore the AoA measurements themselves three sets of tests were presented. The first, and most important, was a controlled experiment with a known array orientation and signal source angle at 900.1 MHz. The second explored the potential of the KrakenSDR to make AoA measurements of multiple signal sources at 900.1 MHz and the third presented some initial testing in the L-band. Results from AoA testing at 900.1 MHz had an average offset from truth of 2.47 degrees with standard deviations for the 90-, 180-, and 270-degree tests being under 2.5 degrees. Considering that the arc-resolution of the array leveraged was ±6 degrees, the results obtained illustrated how the KrakenSDR makes not only accurate measurements well within its expected bounds but does so with consistency. The second set of tests, looking into multiple emitters at 900.1 MHz, offered a look into the ability of the KrakenSDR to identify multiple signal sources. This is limited by the MUSIC algorithm and showed some variation over time but when averaged over the collection period showed promise. Finally, results from a government-sponsored emitter test were briefly presented for the L-band. These results show the potential promise of the KrakenSDR in the L-band but at this stage, due to not being able to verify the testing setup properly, they are primarily used to inform future research into active antenna array calibration, which is succinctly discussed after all of the results.

The results presented in this paper display not only two core components of the KrakenSDR, in its phase calibration and accurate AoA measurements, but its versatility as well. It is our hope that this paper highlights how advancements in radio direction finding hardware are making radio direction finding techniques in radio frequency problems more feasible and affordable. This applies not just to applications in GNSS interference but any other potential problems that may benefit from the use of antenna arrays. Hardware like the KrakenSDR offers the opportunity to approach problems in new ways and thus showcasing this new and novel hardware is paramount to advancing research efforts.

## Figures and Tables

**Table 1:** Full technical specifications of the KrakenSDR [17].

Dimensions	Weight	Typical Power Consumption	Radio Tuner	Radio ADC
177 mm × W: 112.3 mm × H: 25.86 mm	670 g	5 V, 2.2 A (11 W)	5× R820T2	5× RTL2832U
**ADC Bit Depth**	**Frequency Range**	**Maximum Bandwidth**	**Rx Channels**	**Oscillator Stability**
8 bits	24 MHz–1766 MHz	2.56 MHz	5	1 ppm

**Table 2:** UCA arrays designed and used in this work.

Array	*I_e_* [cm]	*s*	Radius [cm]
900 MHz Array (λ = 0.333 m)	11.191	0.336	9.525
L-band Array (λ = 0.187 m)	9.375	0.5	7.969

**Table 3:** Average and standard deviations of phase offsets between channels for calibrated and uncalibrated data.

Phase Difference between Channels	Chn. 1–2 [Degrees]	Chn. 1–3 [Degrees]	Chn. 1–4 [Degrees]	Chn. 2–3 [Degrees]	Chn. 2–4 [Degrees]	Chn. 3–4 [Degrees]	All [Degrees]
Calibrated	6.94 ± 1.29	5.69 ± 1.61	2.29 ± 1.43	1.67 ± 1.19	4.83 ± 1.68	3.58 ± 1.32	4.16 ± 1.42
Uncalibrated	50.03 ± 1.74	15.68 ± 2.05	87.93 ± 2.09	34.35 ± 2.08	137.97 ± 2.12	103.62 ± 1.80	71.60 ± 1.98

**Table 4:** Average and standard deviations of phase offsets between Channel 1 and all other channels for experimental and theoretical results.

Phase Difference between Channels	Chn. 1–2 [Degrees]	Chn. 1–3 [Degrees]	Chn. 1–4 [Degrees]	All [Degrees]
Experimental	137.30 ± 1.29	134.63 ± 1.61	140.33 ± 1.43	137.42 ± 1.66
Theoretical	131.51 ± 1.76	128.94 ± 1.77	134.67 ± 1.05	131.70 ± 1.53

**Table 5:** Mean and standard deviations of AoA and confidence values for single emitter tests [17].

Expected Angle (Deg)	Mean AoA Estimate (Deg)	Mean Confidence Value
0	3.07 ± 19.07	4.73 ± 1.38
90	86.21 ± 2.40	7.37 ± 1.85
180	180.83 ± 1.60	6.29 ± 0.84
270	267.79 ± 1.34	6.10 ± 0.42

**Table 6:** Mean and standard deviations of AoA and confidence values for L1 single emitter tests.

Expected Angle (Deg)	Mean AoA Estimate (Deg)	Mean Confidence Value
0	336.62 ± 19.14	2.26 ± 0.28
90	−11.88 ± 0.59	3.62 ± 0.18
180	192.23 ± 1.18	2.98 ± 0.25
270	261.92 ± 0.48	1.99 ± 0.18

## Data Availability

The data presented in this study are available on request from the corresponding author.
